# Protein Hydrolysis and Glycosylation as Strategies to Produce Bioactive Ingredients from Unmarketable Prawns

**DOI:** 10.3390/foods10112844

**Published:** 2021-11-17

**Authors:** Joaquín Gómez-Estaca, Irene Albertos, Ana Belén Martín-Diana, Daniel Rico, Óscar Martínez-Álvarez

**Affiliations:** 1 Institute of Food Science, Technology and Nutrition (ICTAN, CSIC), 10 José Antonio Novais St., 28040 Madrid, Spain; joaquin.gomez@csic.es; 2 Agricultural Technological Institute of Castile and León (ITACyL), Government of Castile and León, Ctra. de Burgos Km. 119, Finca Zamadueñas, 47071 Valladolid, Spain; irene.albertos@ucavila.es (I.A.); mardiaan@itacyl.es (A.B.M.-D.); ricbarda@itacyl.es (D.R.); 3 Santa Teresa de Jesús Catholic University of Ávila (UCAV), Canteros St., 05005 Ávila, Spain

**Keywords:** protein hydrolysates, Maillard reaction, shrimp by-products, antioxidant, prolyl endopeptidase

## Abstract

The present work shows a procedure to valorize non-commercial boiled shrimp to produce functional ingredients, using a combined treatment based on enzymatic hydrolysis and subsequent glycation under mild conditions. Antioxidant and prolyl endopeptidase-inhibiting activities were determined as a function of hydrolysis and glycation times (0–120 min and 0–180 min, respectively). The reaction products were characterized by determining the degree of hydrolysis, browning, fluorescent compounds, free amino acids, phenol content, Fourier transform infrared spectroscopy (FTIR), and molecular weight of the different fractions obtained. Enzymatic hydrolysis generated hydrolysates with significant antioxidant and prolyl endopeptidase-inhibiting activities. Glycation under mild conditions was used as a strategy to improve the antioxidant and potential nootropic properties of the hydrolysates. During glycation, the free amino acid content decreased, total phenols and fluorescent compounds increased significantly, and low molecular weight melanoidins were formed. The presence of peptide-glucose conjugates was also confirmed by FTIR. Glycation increased the antioxidant activities of the hydrolysates; however, their prolyl-endopeptidase-inhibiting activity was lost. Results showed that compounds with promising antioxidant (hydrolysis and glycation) and potential nootropic (hydrolysis) activities and applications in food systems were obtained from the biotechnological strategy used.

## 1. Introduction

The shrimp market has grown over the last few years as a result of the increasing global demand for crustaceans, leading as well to an increase in industrial shrimp processing and, therefore, in by-products such as heads and shells. Indeed, the global shrimp production reach to 5.03 million tons in 2020 and is expected to grow up to 7.28 million tons by 2025 [1]. These by-products represent approximately 35–45% of the whole shrimp weight [2] and are usually discarded without any attempt of valorization. In addition, no recovery processes have been developed for shrimp that do not meet quality control standards or that expire before sale after long freezing periods [3]. Furthermore, these by-products represent an environmental hazard, and their management constitutes a considerable cost for the industry. Therefore, it is necessary to investigate viable processes to valorize this large amount of shrimp by-products, which, in turn, are highly rich in protein [4]. The use of proteases has been reported as an efficient biotechnological strategy to obtain protein hydrolysates with technological and nutraceutical applications [4,5,6]. However, it should be noted that the properties of the hydrolysates obtained depend largely on the proteases and raw materials used as well as on the hydrolysis conditions.

Peptides resulting from protein hydrolysis can exhibit interesting antioxidant properties [7] (Li-Chan 2015). Guerard et al. [6] highlighted the important influence of pH and temperature in the production of antioxidant peptides from the hydrolysis of shrimp processing discards. Ketnawa et al. [3] obtained an antioxidant protein hydrolysate from cooked shrimp that was incorporated into fish tofu and thus delayed lipid oxidation and microbial spoilage. In addition, peptides with an inhibitory effect against prolyl endopeptidase (also known as prolyl oligopeptidase, PEP) and dipeptidyl peptidase-IV (DPP-IV) have been obtained by the hydrolysis of shrimp protein residues [5]. Peptides showing prolyl endopeptidase (PEP) inhibitory activity could be of interest as nootropic ingredients in functional foods, as altered PEP levels are associated with neuropathological disorders and different types of dementia such as Alzheimer’s disease [8].

The Maillard reaction (or Maillard glycation) proceeds in three different steps (early, intermediate, and advanced). The first step involves a non-enzymatic reaction between the nucleophilic amino groups of amino acids, peptides, or proteins, and the carbonyl groups of reducing sugars, to produce a glucosylamine that spontaneously undergoes the Amadori rearrangement, eventually forming derivatives called Amadori products [9,10]. Many Maillard reaction products (MRPs) such as ketones, dicarbonyls, furans, aldehydes, and others are formed in the intermediate step, while brown molecules, fluorescent compounds, and cross-linked polymers are generated in the advanced step [10,11]. The complex variety of MRPs formed depends on the source of reactive amino groups, the type of reducing sugar, and the technological conditions used. Some of these MRPs may have detrimental health effects, while others may exert beneficial antimicrobial and antioxidant effects [11,12,13,14,15,16]. The development of the Maillard reaction under controlled conditions can prevent the generation of advanced glycation products (AGEs), which are implicated in different pathologies such as diabetes mellitus, atherosclerosis, Alzheimer’s disease, dialysis-related amyloidosis, and the aging process [17].

The controlled Maillard reaction has been successfully used to improve the antioxidant activity of fish waste hydrolysates [14,18]. However, little is known about the effect of the Maillard reaction on other functional or bioactive properties. According to Horvat and Jakas [13], the nature of the products formed in the early stages of the Maillard reaction will depend on the structure of peptide moieties and the length of peptide chains in the system, considering that the reactivity of the sugar is also important [19]. Protein hydrolysates contain a wide variety of peptides with amino groups capable of reacting with carbonyl groups; thus, the Maillard reaction will form a very heterogeneous group of MRPs with technological and/or biological properties that can be improved from those of the original hydrolysate. Recently, Djellouli et al. [20] obtained antioxidant MRPs from the controlled reaction of a shrimp protein hydrolysate with glucosamine. Cai et al. [21] explored the production of flavoring agents by reacting shrimp waste hydrolysates with xylose at 115 °C and observed differences in flavor as a function of heating time. They suggested that these products could have interesting applications in food. Despite these promising results, the reality is that the number of studies that have addressed this combined process of hydrolysis (especially of seafood protein) followed by mild glycation to obtain ingredients of technological and/or functional interest is very limited.

The main objective of this work was to explore new strategies to valorize shrimp processing by-products by obtaining compounds with antioxidant and/or PEP-inhibiting activities from the reaction of shrimp by-product hydrolysates with glucose under mild conditions. While enzymatic hydrolysis can yield molecules of antioxidant or nootropic interest, the aim of the Maillard reaction was to enhance these activities and thereby obtain molecules with greater potential as bioactive and/or technological ingredients for the food industry.

## 2. Materials and Methods

### 2.1. Chemicals

2,2′-Azinobis 3-ethylbenzothiazoline-6-sulfonic acid (ABTS•+), 2,2′-diazobis-(2-aminodinopropane)-dihydrochloride (AAPH), fluorescein, 2,2-diphenyl-1-picrylhydrazyl (DPPH), Folin–Ciocalteu (FC) reagent, gallic acid (GA), o-phthaldialdehyde reagent (OPA reagent), 6-hydroxy-2,5,7,8-tetramethyl-2-carboxylic acid (Trolox), and glucose were from Sigma-Aldrich, Co. (St. Louis, MO, USA). Prolyl endopeptidase (PEP) from Flavobacterium was from Seikagaku Corp. (Tokyo, Japan). Z-Gly-Pro-7-amido-4-methylcoumarin was from Bachem. The commercial enzyme preparation Alkaline protease from *Bacillus licheniformis* (activity of 2,000,000 DAPU/g, optimum pH 7–10, optimum temperature 50–60 °C) was kindly provided by Bio-Cat Inc. (Troy, VA, USA). Other chemicals were of analytical reagent grade.

### 2.2. Preparation of Shrimp Hydrolysate and Maillard Glycation

Cooked shrimp (*Penaeus vannamei*) were stored at −20°C for 24 months and thawed overnight before testing. The protein hydrolysate was obtained according to the protocol described by Ketnawa et al. [5] with slight modifications. Five hundred grams of peeled shrimp were minced in a blender for 20 s. Then, the minced samples were mixed with 700 mL of 0.1 M phosphate buffer, pH 8. Before hydrolysis, the pH was adjusted to 8 with 1 M NaOH. Proteins were digested using 3 g (460 units) of alkaline protease, considering 1 unit as the amount of enzyme required to release 1 µmol Tyr/min under the experimental conditions used [22]. The hydrolysis was carried out in a batch reactor of 2 L at 50 °C. The pH was maintained at the desired value (8) using a pH-stat TIM 856 (Radiometer Analytical, Villeurbanne, France). Different aliquots were collected at minutes 10 (H10), 30 (H30), 60 (H60), and 120 (H120) of the reaction. A control sample without alkaline protease was heated at 50 °C for 120 min. The reaction was stopped by heating the solutions at 90 °C for 20 min in a thermostatic bath (Selecta Unitronic OR, Barcelona, Spain). The samples were filtered through two layers of cheesecloth to separate large particles and further centrifuged at 14,000× *g* at 5 °C for 30 min (Beckman Coulter J2-mc, Indianapolis, IN, USA). The supernatants were collected and stored at 4 °C until use.

The Maillard glycation was carried out according to Djellouli et al. [20]. Both the control and shrimp hydrolysates were first mixed with glucose (2:1, *v*:*w*) in screw-capped glass tubes. Then, the pH was adjusted to 8.4. The samples were heated to 100 °C in a temperature-controlled water bath with stirring (120 rpm). Different aliquots were collected at minutes 0, 40, 60, 120, and 180, immediately cooled on crushed ice, and freeze-dried for 72 h in a lyophilizer (VirTis model Benchtop-6KB, Zaragoza, Spain).

### 2.3. Molecular Weight Distribution

The molecular weight (Mw) profile of each hydrolysate before and after glycation was measured by size exclusion chromatography, as described by Lajmi et al. [22], with modifications. A Superdex 30 Increase 3.2/200 column from GE Health-care Bio-Sciences (Barcelona, Spain), with bed dimensions of 3.2 × 300 mm, was connected to an HPLC consisting in an injector (model SIL-10AD vp), a pump (model LC-10AD vp), an UV-Vis detector (model SPD-10A vp), a column oven (model CTO-10AC vp), and a system controller (model SCL-10A vp), all from Shimadzu (Tokyo, Japan). The mobile phase consisted of 30% (*v*/*v*) acetonitrile with 0.01% (*v*/*v*) TFA. The flow rate was 750 µL/min and the injection volume was 5 µL. The absorbance of the hydrolysates was read at 214 nm, while that of the glycated samples was read at 280 and 420 nm. The Mw of the most abundant populations was calculated based on the elution time of the following standards: bovine serum albumin (BSA, 6700 Da), polylysine (4700 Da), vitamin B12 (1340 Da), hippuryl-L-histidyl-L-leucine (429 Da), and glycine (75 Da). The relative amount of the most abundant molecules was calculated by measuring the height of the corresponding peaks and was expressed as a percentage of the total. The Mw distribution in the ranges 10–1 kDa, 1–0.5 kDa, and below 0.5 kDa was calculated by measuring the areas in the chromatogram and was expressed as a percentage of the total.

### 2.4. Determination of Protein Content

The nitrogen content in the solid shrimp waste was determined with a LECO FP-2000 nitrogen analyzer (LECO Corp., St. Joseph, MI, USA), previously calibrated with EDTA, according to Lajmi et al. [22]. The total protein content was calculated using a nitrogen-to-protein conversion factor of 6.25. The protein content in the solid waste was expressed as percentage (g/100 g).

The protein content of the liquid samples resulting from the heating reaction of the protein hydrolysates with glucose was quantified using the Bradford assay and expressed as g/100 mL. Bovine serum albumin (BSA) at concentrations of 0.1–0.6 mg/mL was used as a standard. The correlation coefficient for the standard calibration curve was 0.99.

### 2.5. Degree of Hydrolysis (DH), Free Amino Acids, and Browning

The degree of hydrolysis (DH) was defined as the percentage of cleavage peptide bonds relative to the total number of peptide bonds, and it was quantified according to Ketnawa et al. [5], using the next formula:

$$DH=\left(\frac{B\times Nb}{Mp\times \alpha \times htot}\right)\times 100$$

*B* was the amount of NaOH used to keep the pH constant during the protein hydrolysis; *Nb* was the normality of the NaOH used; *Mp* was the protein content in the shrimp waste (defined as N × 6.25), α was the average degree of dissociation of the α-NH_2_ groups released during the hydrolysis, and *htot* was the total number of peptides bonds/protein equivalent. The results were expressed as percentage of peptide bonds released.

The amount of total free amino acids before and after the reaction with glucose was calculated and measured using OPA (O-phthalaldehyde) according to Nielsen et al. [23]. An aliquot of 10 µL of each sample and standard (Serine) was mixed with 100 µL of freshly prepared OPA reagent in a 96-well microplate. It was incubated at room temperature for 5 min, and absorbance readings were taken at 340 nm using a microplate reader Fluostar Omega (BMG, Ortenberg, Germany). Milli-Q water was used as the blank. The mean value of the absorbance readings obtained from three assays was used for the calculations. The DH was calculated using the equation described by Nielsen et al. [23]. The results were expressed as mEq Ser NH_2_/g of protein.

The browning intensity of the samples at different glycation times was quantified by reading the absorbance at 420 nm on an Appliskan Multimode microplate reader (Thermo Fisher Scientific, Waltham, MA, USA), according to Djellouli et al. [20]. The fractions were pre-diluted with distilled water according to the initial color intensity. The degree of dilution was taken into account to calculate the final browning value of each sample. The results were expressed as the increase (%) relative to browning at time 0.

### 2.6. Measurement of Fluorescence

The increase in fluorescence throughout the Maillard reaction was determined as a measure of the formation of intermediate molecules, such as Amadori compounds, according to Djellouli et al. [20]. The samples collected at different glycation times were firstly diluted in 20 mM phosphate-buffered saline (pH 7, 15 mM NaCl), and then, fluorescence was measured at λexc 340 nm and λem 410 nm in an Appliskan Multimode microplate reader. The excitation wavelength for maximum emission was determined in a previous experiment.

### 2.7. Total Phenols (TPs)

TPs were quantified in all the samples, included the controls, using the Folin-Ciocalteu method as described by Slinkard and Singleton [24], with modifications. A volume of 140 µL of the sample extract was mixed with 280 µL of Folin–Ciocalteu reagent previously diluted (1:10, *v*/*v*) and 980 µL of 42.86 mM sodium carbonate. The mixture was shaken and allowed to stand for 100 min in darkness, following centrifugation at 15,000× *g* for 3 min. The absorbance was measured at 765 nm with a microplate reader Fluostar Omega. The results were expressed as mEq gallic acid (GAE)/100 g of sample (dry basis) using a calibration curve with gallic acid as a standard (9.8–700 µM). The correlation coefficient for the standard calibration curve was 0.99. The samples were evaluated in duplicate.

### 2.8. Fourier Transform Infrared Spectroscopy (FTIR)

The FTIR spectra of the samples before and after the reaction with glucose were obtained in a Perkin-Elmer Spectrum 400 Infrared Spectrometer (Perkin-Elmer Inc., Waltham, MA, USA) equipped with an ATR prism crystal accessory. The spectral resolution was 4 cm^−1^. Measurements were performed at room temperature using approximately 1 mg of dried sample, which was placed on the surface of the ATR crystal and pressed with a flat-tip plunger until spectra with suitable peaks were obtained. Background interference was eliminated using the Spectrum software version 6.3.2 (Perkin-Elmer Inc., Waltham, MA, USA).

### 2.9. Antioxidant Activity

#### 2.9.1. ABTS•+ Radical Cation Scavenging Activity (ABTS•+)

The classical version of this method was adapted from the assay developed by Martin-Diana et al. [25]. A stock ABTS·+ solution was prepared by mixing a 7 mM aqueous ABTS solution with 2.45 mM K2O8S2 in a 1:1 (*v*/*v*) ratio. Before the assay, the stock ABTS·+ solution was diluted with phosphate buffer (75 mM, pH = 7.4) to obtain a working solution with an absorbance value of 0.70 ± 0.02 at 734 nm. Then, a volume of 20 µL of diluted samples was mixed with 200 µL ABTS•+ working solution in a 96-well microplate. The decay in absorbance at 730 nm was monitored over 30 min with a microplate reader. A calibration curve with Trolox (7.5–240 μM) diluted in the extracting solvent was used as standard (correlation coefficient of 0.99). The results were expressed as µEq Trolox/100 g sample (dry basis).

#### 2.9.2. Oxygen Radical Absorbance Capacity (ORAC)

The procedure was based on the method previously described by Ou et al. [26], with modifications. The samples were diluted in phosphate buffer (10 mM, pH 7.4). Fluorescence was monitored over 150 min with a microplate reader Fluostar Omega at λexc 485 nm and λem 520 nm. Trolox (7.5–240 mM) was used as a standard. The results were calculated using the areas under the fluorescence decay curves, which were blank corrected (run without antioxidants) and compared to those areas obtained using Trolox standard concentrations (7.5–180 µM). The correlation coefficient for standard calibration curve was 0.99. The results were expressed as µEq Trolox/100 g sample (dry basis).

#### 2.9.3. DPPH· Radical Scavenging Activity

The extract-based DPPH· assay was performed as described by Brand-Williams et al. [27] with modifications. A 120 µM DPPH·working solution in pure methanol was prepared. In a 96-well microplate, 25 µL of the extracts were mixed with 100 µL of Milli-Q water and 125 µL of DPPH working solution. The decay in absorbance at 525 nm was recorded over 30 min with a microplate reader Fluostar Omega. Different solutions of Trolox (7.5–240 µM) were evaluated to perform a calibration curve. The correlation coefficient for the standard calibration curve was 0.99. The results were expressed as µEq Trolox/100 g sample (dry basis).

#### 2.9.4. Ferric-Reducing Antioxidant Power (FRAP)

A FRAP assay was performed following the protocol reported by Benzie and Strain [28]. The FRAP reactive was composed of acetate buffer (300 mM, pH 3.6), TPTZ (10 mM), and FeCl_3_ (20 mM) as a ratio of 10:1:1, respectively and kept at 37 °C. One hundred μL of the extracts, 1.8 mL of FRAP reactive, and 1.2 mL of distilled water were mixed and incubated at 37 °C for 15 min. Then, the absorbance was measured with a microplate reader at 593 nm. FeSO_4_·7H_2_O was used as the standard (2–4 mM). The correlation coefficient for the standard calibration curve was 0.99. The results were expressed as mEq Fe/100 g sample (dry basis).

### 2.10. Prolyl Endopeptidase (PEP)-Inhibiting Activity

The PEP inhibitory activity of each hydrolysate before and after the reaction with glucose was measured according to Sila et al. [29]. The samples were diluted in 0.1 M sodium phosphate buffer and tested for the PEP-inhibiting ability in 96-well microplates. In brief, 20 µL of enzyme (1 mU) was mixed with 30 µL of sample and 150 µL of assay buffer and incubated for 15 min at 30 °C. Then, 100 µL of substrate (0.01 mM Z-Gly-Pro-AMC) were added, and the increment in fluorescence (λexc 340 nm and λem 450 nm) was measured at 1 min intervals for 20 min using the microplate reader. Six controls were used, using the assay buffer instead of sample. The inhibitory activity of the samples (final concentration in the 1 mg/mL system) was calculated from the maximal increment in fluorescence in the presence or absence of sample and was shown as a percentage of inhibition.

### 2.11. Statistical Analysis

The statistical analyses were performed using Statgraphics Centurion XVI. All analyses were carried out in triplicate. The experimental data were subjected to analysis of variance (one-way ANOVA). A MANOVA (multivariate analysis of variance) test was also used to find any inter-relationships between hydrolysis and glycolysis time. The differences between pairs of means were assessed based on confidence intervals using Fisher’s LSD (Least Significant Difference) with a level of significance of *p* < 0.05.

## 3. Results and Discussion

### 3.1. Effect of Reaction Time on DH and Free Amino Acid Content of Shrimp

The DH increased rapidly in the first ten minutes up to 6.07% due to the large number of peptide bonds available. After 60 min, a significant reduction in the reaction rate was observed (DH = 12.07%), which finally remained constant until the end of the process (Table 1), which was probably due to the lower amount of substrate available, enzyme self-digestion, and product inhibition [30]. After 2 h of hydrolysis, the DH was 12.08%, this value being higher than that obtained by Ketnawa et al. [5] after the hydrolysis of cooked shrimp, using different proteases. These variations could be attributed to the different proteases used as well as to the heat-induced protein denaturation, which could affect the accessibility of proteases to peptide bonds.

The free amino acid content increased during the hydrolysis time, reflecting the activity of alkaline protease (Table 1). After glycation, a general decrease in the free amino acid content of the samples was observed. Although the amino acid profile of the glycated and non-glycated samples was not determined, it may be assumed that the significant decrease in free amino acid content (16–48%) is the result of the reduction of available primary amino groups caused by the reaction with the reducing carbonyl groups of glucose [19]. In previous work, Djellouli et al. [20] observed a significant decrease in lysine and arginine contents after the reaction of Pacific white shrimp protein hydrolysates with glucosamine. These authors also observed a decrease in the amount of glutamic acid and glutamine, glycine, valine, and isoleucine, among other amino acids, which could have reacted with glucose if located at the N-terminal position of some peptides. Nonetheless, it cannot be ruled out that prolonged heating in the presence of glucose may have induced some thermal degradation of specific amino acids in the glycated samples [31]. An unexpected increase in the total amount of free amino acids was observed after the glycation of H30. Similar increases were observed by Djellouli et al. [20] but in the absence of reducing sugars, and these could be attributed to peptide hydrolysis caused by prolonged heating [32]. The degradation of peptides in this sample may occur at the same time as the reaction with glucose, which should result in different MRPs than would be expected.

### 3.2. Evolution of the Average Molecular Weight (Mw) during Protein Hydrolysis

The average Mw decreased over the hydrolysis time mainly due to the release of peptides (Table 2). This was reflected in the relative amount of peptides found with a Mw below 500 Da, which increased over time, and also of those in the 500–1000 Da range, which highly augmented during the first 10 min. Peptides with Mws around 574 Da, followed by others around 279 and 949 Da, were predominant in the sample that was hydrolyzed for 10 min. From then on, the protein was scarcely hydrolyzed, reaching a predominant Mw of 857 Da. The average Mw of other predominant peptides (around 570–580 and 270–280 Da) remained relatively constant until the end of the hydrolysis.

### 3.3. Evolution of the Average Molecular Weight during the Maillard Reaction

In the glycated samples, absorbance at 214 nm was employed to determine the presence of molecules with amide bonds in their structure. As well, to detect molecules with aromatic rings and browning compounds, absorbance measurements at 280 nm and 420 nm were obtained, respectively. It is important to note that the protein hydrolysates contained a very diverse pool of amino acids and peptides that could produce a very complex mixture of molecules after the Maillard glycation.

During the first 40 min of the reaction with glucose, the control sample mainly yielded molecules with Mws around 200 and 335 Da, along with other molecules of around 600 Da (Table 2). This Mw profile, rich in low Mw molecules (67–69%), remained constant until the end of the reaction. In addition, molecules of around 6500–7000 Da, presumably polymers derived from the interaction of glucose with the soluble polypeptides present in the sample, were found.

The products of the reaction of the protein hydrolysates with glucose showed different Mw profiles as compared with the one observed in the control sample. In almost all cases, low Mw molecules predominated, mainly those of around 350 Da, in some cases reaching up to 70–79% (Table 2). These molecules were formed during the first 40 min and were likely to remain stable throughout the Maillard reaction. Molecules of around 230–260 Da, apparently dipeptides and/or the result of the reaction of glucose with short dipeptides or free amino acids, were also abundant (approximately 30%). Moreover, molecules of 700–800 Da were found in all cases (about 10–20%), with certain changes in their Mw being observed during the heating reaction. The slight differences observed when the reaction times are the same, and which depend on the hydrolysate used, indicate the influence of peptide composition in the formation of these molecules. Considering the difference in terms of molecules with a Mw of around 850–950 Da as compared with the hydrolysates, it seems that this peptide population played an important role in the generation of these new 700–800 Da molecules in the reaction with glucose, whereas the other populations most likely participated in the formation of the lowest Mw molecules.

In all cases, the most abundant molecules found at 214 nm were also predominant at 280 nm (Table 2), indicating the presence of an aromatic ring in their structure. These molecules could be the result of the Strecker reaction during the first 40 min of heating. The proportion of molecules below 500 Da detected at 280 nm was significantly high (65–91%), which indicates that aromatic molecules were mainly formed during the first minutes of the reaction. However, this proportion decreased slightly thenceforward, suggesting the generation of aromatic polymers.

Interestingly, the high Mw molecules derived from the reaction of the control sample with glucose detected at 214 nm (Table 2) were not found at 280 nm (Table 2). The molecules with a Mw above 1 kDa only accounted for a maximum of 8% of the relative abundance. Furthermore, the most abundant molecules found at 280 nm were very different from those detected at 214 nm, indicating that the reaction of glucose with proteins or peptides followed different pathways.

### 3.4. Evolution of Browning during the Maillard Reaction

**Figure 1:**
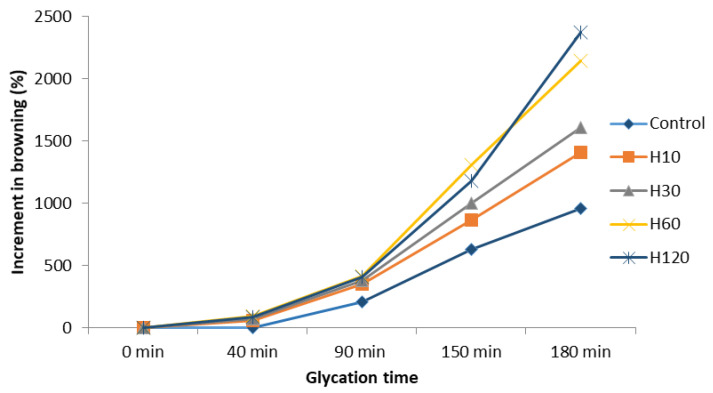
Increment in browning during the Maillard glycation of the control and the hydrolysates. The increments were calculated from the browning of each sample at time 0.

Browning is the result of melanoidin formation and was studied in this work as a measurable parameter indicating the extent of the Maillard reaction. Browning intensity was similar in all samples during the first 40 min of the reaction, increasing significantly thereafter, mainly when H60 and H120 were used as a source of amino groups (Figure 1). Although heated glucose was not used as a control, sugar caramelization is not probably involved in the increased browning, as observed by Laroque et al. [19] and Morales and Jiménez-Pérez [10]. Whereas melanoidins can have Mws higher than even 100 kDa [11], the Mw profiles obtained at 420 nm (Table 2) indicated that the increment in browning was mainly produced by the formation and accumulation of low Mw brown-colored compounds, as previously reported [11,33].

The browning intensity of the control sample increased over time, mainly after 150 min of heating, and it was concurrent with changes in the composition of low Mw melanoidins (Table 2). Molecules of around 350 Da were formed during the first 90 min but from then on, they disappeared, which was probably due to condensation processes. In addition, a small quantity of melanoidins with Mws above 1 kDa (4–11%) was produced during glycation.

Low Mw melanoidins (below 500 Da) were found to be the main components of the glycated hydrolysates (Table 2); however, their composition changed over time. During the first 90 min, melanoidins of around 280–300 Da were predominant. From then on, other molecules of 500–600 Da were formed, but in a smaller proportion (12–16%). As well, a slight tendency to form melanoidins with higher Mws than 1 kDa was observed (10–20%), coinciding with a sharp increase in browning intensity. This tendency to increase the average Mw during glycation has been previously described [34]. Kim and Lee [15] found that Mw increased markedly with heating time during the reaction of glucose with glycine, diglycine, and triglycine. The slight presence of melanoidins with higher Mws than 1 kDa and the absence of those with higher Mws than 10 kDa (data not shown) suggest that the new products formed hardly polymerize and hence are not the result of the reaction of intermediate molecules with reactive amino groups that is observed in advanced states of the Maillard reaction [11]. Interestingly, the higher the Mw of the larger molecules and their relative content, the lower the DH of the samples. These results indicate that a higher amount of low Mw peptides is associated with a higher production and accumulation of brown compounds, which could be ascribed to the presence of a greater number of amino groups susceptible to react with glucose in the most hydrolyzed samples (Table 1). These results are in agreement with Kim and Lee [15].

The low Mw melanoidins were presumably generated by the reaction of Amadori compounds with amino acids [11,17,35] or by the reaction of amino acids with conjugated enediols (i.e., reductones) produced by the enolization of Amadori compounds and their subsequent dehydration. Djellouli et al. [20] observed a different trend in browning during the first 40 min when heating a shrimp protein hydrolysate with glucosamine at 100 °C. This indicates that the induction periods are different based on the type of sugar used, as previously proposed [19]. Hofmann [33] also reported the predominance of colored compounds of Mws below 1000 Da in glucose/amino acid mixtures heated at 95 °C for 4 h and observed the presence of low amounts of brown molecules of 1000–3000 Da. Wang et al. [11] reviewed the structure of melanoidins and reported the production of low Mw melanoidin-type compounds containing furan, pyrrole, and pyrrolinone structures and derivatives by heating xylose/glucose and alanine/proline/lysine for 5 h.

### 3.5. Evolution of Fluorescence during the Maillard Reaction

The increase in fluorescence along the Maillard reaction has been associated with the formation of intermediate products, such as Amadori compounds [13,36]. The fluorescence of the samples was firstly measured at different excitation and emission wavelengths (data not shown). The maximum fluorescence, in all cases, was obtained at 340 and 410 nm, respectively. This suggests a common formation pathway for fluorescent compounds. The maximum excitation wavelength was in agreement with that reported by Matiacevich et al. [37] for fluorescent products of the Maillard reaction (340–370 nm). In contrast, the maximum emission wavelength was lower than that registered by these authors (420–450 nm). Djellouli et al. [20] described different optimum excitation and emission wavelengths for the compounds formed during the reaction of shrimp protein hydrolysates with glucosamine. This is probably attributable to different variables such as the type of protein and carbohydrate sources, and reaction conditions in terms of pH and temperature, for instance.

**Figure 2:**
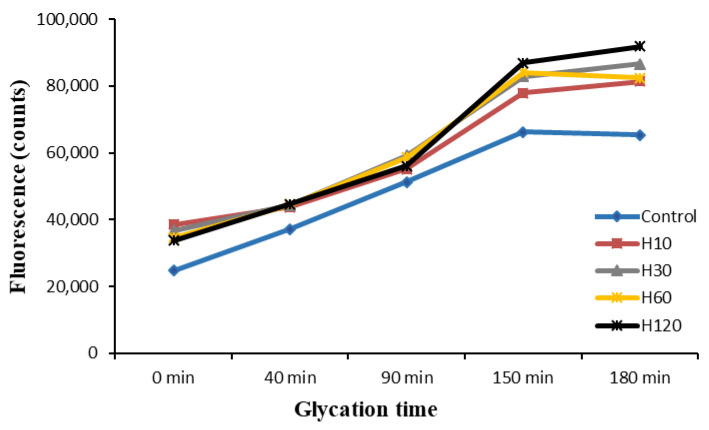
Fluorescence of the samples along Maillard glycation.

The fluorescence of the glycated hydrolysates increased slightly during the Maillard glycation (Figure 2) in contrast to the significant increases observed by Djellouli et al. [20] during the reaction of shrimp protein hydrolysates with glucosamine. Morales et al. [36] studying different model systems and milk also noted that fluorescent compounds were formed during heating. The highest increase was observed during the glycation of H120, which was probably due to the higher reactivity of low Mw peptides, as previously observed by Su et al. (2011). According to Horvat and Jakas [13], the presence of low Mw peptides is associated with a higher degradation of Amadori compounds, as well as with the development of Maillard fluorescence. Interestingly, a slight decrease in fluorescence was observed in all hydrolysates after 90 min of reaction with glucose. This coincided with changes in the molecular weight profiles observed at 214 nm (Table 2), and it could be the result of the disappearance and creation of intermediate molecules of different fluorescence intensity.

A considerable accumulation of fluorescent intermediate molecules was observed from the first minutes of glycation of the control sample before brown pigments were significantly generated (Figure 1). This suggests that these fluorescent intermediates could be precursors of the low Mw melanoidins formed in more advanced steps [10,21,36,38]. From min 40 to 150 of the reaction, fluorescence increased dramatically together with the increase in browning, indicating the concomitant formation of fluorescent and colored intermediate MRPs. During the last 30 min of reaction, in all samples, fluorescence either suffered a slight increase (H10, H30, H120) or remained stable (control and H60), suggesting that the fluorescent intermediates are rather stable upon prolonged heating.

### 3.6. Phenol Content

The phenol content in the protein hydrolysates was significantly higher (*p* < 0.05) than that of the control sample (Table 3). This is probably due to soluble peptides with tyrosine residues being released from insoluble muscle proteins during the hydrolysis process. The phenol content increased significantly (*p* < 0.05) until 30 min of hydrolysis, and no peptides with tyrosine residues were released thereafter.

During the Maillard glycation, the phenol content increased significantly (*p* < 0.05) in all cases, regardless of the peptide source used (Table 3), which was probably as a result of Amadori rearrangements. However, the molecular profiles observed at 280 nm did not indicate relevant changes in the phenolic compound composition of the samples analyzed, suggesting that the significant differences observed at the same glycation times were the result of the formation of aromatic molecules at different concentrations. Moreover, the results seem to indicate that there is no relationship between phenol content and DH.

### 3.7. Fourier Transform Infrared Spectroscopy (FTIR)

**Figure 3:**
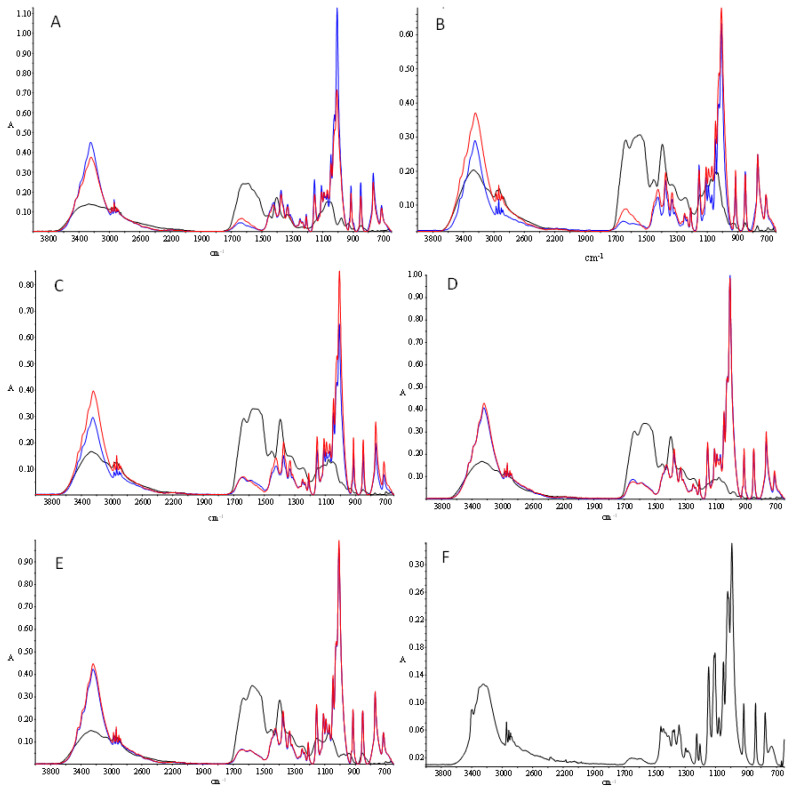
FTIR spectra of control (**A**), H10 (**B**), H30 (**C**), H60 (**D**), and H120 (**E**) samples. Black, blue, and red lines represent the non-glycated samples and the samples subjected to reaction with glucose for 40 min or 180 min, respectively. Figure (**F**) represents the spectrum of glucose.

In the spectra (Figure 3) of the hydrolysates and the control sample, several characteristic protein absorption bands were observed, such as the amide A, amide I, and amide II bands, typically found at ≈3300 cm^−1^, 1600–1690 cm^−1^, and 1480–1575 cm^−1^, respectively [39] (Figure 3). Other characteristic protein bands were not observed, which was probably due to the inability of peptides to adopt complex conformations in contrast with native proteins. The amide A band, which is ascribed fundamentally to NH stretching vibrations, could be seen at ≈3265 cm^−1^ in all samples. The amide I band was observable at 1634 cm^−1^ in hydrolyzed samples (Figure 3B–E). This band, mainly attributable to C=O stretching vibrations and, to a lesser extent, to NH in-plane bending vibrations of the peptide linkages [39], is very sensitive to protein conformational changes. The amide II band appeared in the range 1538–1575 cm^−1^, depending on the sample. This band derives primarily from NH in-plane bending but also from CN stretching vibrations [39]. In the case of the control sample (Figure 3A), a clear resolution between amide I and amide II bands could not be observed, showing a maximum al ≈1590 cm^−1^. This is probably due to protein aggregation produced by boiling and prolonged freezing. However, during hydrolysis, peptide fragments are released, and their secondary structures can be observed. This is supported by the fact that amide I and II band intensities increased proportionally with hydrolysis time. It is also worth noting the marked intensity peak found in the band at 1400–1450 cm^−1^ (attributed to C-H bending modes) in the hydrolyzed samples, as compared with the control sample, indicating the presence of peptides [40].

The spectrum of glucose, which was determined for comparative purposes, showed some peaks as it is described in the literature [41] (Figure 3F). In carbohydrates, the most intense bands appeared in the region 1180–953 cm^−1^, resulting from vibration modes such as CC and CO stretching and the bending mode of CH bonds [42]. The reaction of the different samples (hydrolysates and control) with glucose gave rise to evident changes in the spectra; however, only minor differences were found based on the type of hydrolysate (hydrolysis time) and on the reaction time with glucose. In the region 3600–3200 cm^−1^, the wideband corresponding to NH stretching vibrations in proteins (amide A) showed a very marked intensity increase after reacting with glucose, and the maximum peaks shifted toward lower wavelengths (≈3239–3249 cm^−1^), which was probably as a consequence of the contribution of OH stretching vibrations [43,44]. This is indicative of the formation and accumulation of melanoidins [20,43] and is in line with the increment in browning (Figure 1). However, a clear relationship between the increase in band intensity and glycation time was not observed in all samples. The sharp and well-defined peaks detected at ≈2969 cm^−1^ and 2935 cm^−1^ are attributed to –CH3 and –CH2 and have been previously observed in melanoidin samples [43].

After the reaction with glucose, the amide I and amide II bands disappeared completely in all samples, even after the shortest treatment (40 min). At the same time, a new band at 1641–1642 cm^−1^ appeared, which is compatible with C=N stretching vibrations. Both events are indicative of the implication of proteins in the formation of Schiff bases with glucose as a result of the Maillard reaction [45]. Other authors evidenced a reduction of the intensity of amide I and amide II bands in systems composed of soy protein/soy soluble polysaccharides or casein/glucose, which was associated with the formation of Maillard reaction products [46,47]. The intense bands found in the 1200–900 cm^−1^ region in the glycated samples are the typical bands associated with glucose, indicating that many functional groups of glucose remain intact.

### 3.8. Prolyl Endopeptidase-Inhibiting Activity (PEP)

The protein hydrolysates showed PEP inhibitory activity with a maximum value of 40.9 ± 1.9% after 10 min of hydrolysis (H10). Thereafter, the inhibitory activity decreased significantly (20.8 ± 3.4%) and remained constant until the end of the hydrolysis, reaching values close to 25.3 ± 4.1%. Thus, the most active PEP inhibitory peptides were formed during the first minutes of the reaction but were subsequently hydrolyzed by alkaline protease. The PEP inhibitory activity of H10 showed similar values to those previously reported in cooked shrimp hydrolysates prepared with trypsin or alkalase [5]. However, while these authors hydrolyzed shrimp protein for 3 h, in the current work, 10 min were enough to obtain similar results. Sila et al. [29] also obtained comparable results in hydrolysates from barbell skin gelatin prepared with different enzymes, although the average Mw reported was much higher than 1000 Da. On the contrary, Lajmi et al. [22] obtained PEP-inhibitory hydrolysates rich in a wide variety of peptides below 500 Da from smooth-hound by-products, which indicates that the inhibition is likely related to the presence of specific sequences or certain amino acids located at particular positions in the peptide chain.

The heating reaction with glucose led to the loss of PEP inhibitory activity, at least at the concentration tested in this study, suggesting that the Maillard reaction caused important changes in the structure of the inhibiting peptides, which modified negatively their binding to the enzyme molecule. Nonetheless, the modification in the peptides structure produced by prolonged heating times should not be dismissed.

### 3.9. Antioxidant Activity

All samples showed antioxidant activity regardless of their DH, as shown in Table 3. The antioxidant activity was significantly improved (*p* ≤ 0.05) owing to protein hydrolysis, except for DPPH radical scavenging activity, as determined by the antioxidant tests performed. The antioxidant activity of the different hydrolysates at equal glycation time was in almost all cases significantly different, although no clear trend was observed. The highest antioxidant activity was observed in samples H10, H30, and H60, depending on the technique used and the glycation time. These results are consistent with those presented by Guerard et al. [6], who suggested that the antioxidant capacity depends on the composition, structure, and hydrophobicity of the peptides present in the hydrolysates [48,49], rather than on the Mw of the peptides. Additionally, a wide variety of antioxidant compounds present in the hydrolysates, such as astaxanthin, histidine dipeptides (e.g., anserine, carnosine), and free amino acids (aspartic acid, glutamic acid, alanine, leucine, lysine, and taurine), could also have participated in the antioxidant activity.

The reaction of the hydrolysates with glucose for at least 40 min significantly (*p* ≤ 0.05) increased the antioxidant capacity of all samples (Table 3) as compared with that of the control. This increase was correlated with a slight increment in browning and is attributed to the formation of fluorescent intermediates and low Mw melanoidins [10,11]. However, the strong increase in browning during the first 150 min of reaction was not clearly associated with an increase in antioxidant activity, which is consistent with the lack of positive correlation between radical scavenging activity and browning previously described [10,50,51]. On the contrary, all the antioxidant activities analyzed increased markedly (*p* < 0.05) during the last 30 min of the reaction, coinciding with a strong increase in browning and the appearance of low Mw melanoidins (Table 2). These molecules are probably responsible for the strong antioxidant activity, as suggested by Wang et al. [11] and Murakami et al. [51], implying that not all the colored compounds generated during the reaction exerted the same antioxidant activity. Interestingly, the antioxidant activity of the samples that were glycated for 180 min was higher when the average Mw of the protein hydrolysate used was lower, indicating that the smallest peptides play an important role in the generation of colored compounds with strong antioxidant capacity, as observed by Su et al. [52].

The free radical scavenging activity, reducing power, and metal-chelating capacity of melanoidins have been previously reported by different authors [10,11,53,54]. The aforementioned could be attributed to their metal-chelating ability, their capacity to reduce hydroperoxides and to break the free radical chain reaction by donating hydrogen, and/or their ability to scavenge hydroxyl radicals [11,55]. Other molecules with reducing power, such as reductones or glucose degradation products, could also be formed under alkaline conditions and contribute to the antioxidant activity [54,56].

## 4. Conclusions

This work shows that a combined treatment based on protein hydrolysis and a subsequent glycosylation reaction under mild conditions is an effective strategy to valorize uncommercial cooked shrimp since it generates compounds with antioxidant activity and with interesting applications as a natural antioxidant to preserve food quality during storage. A process consisting of 120 min of protein hydrolysis followed by 180 min of glycation under mild conditions will produce the most potent antioxidant molecules from the shrimp waste. The hydrolysis time could be shortened to 10 min due to its higher energy efficiency, although the antioxidant activity after glycation would be somewhat lower than that of the shrimp waste hydrolyzed for 120 min and glycated. Moreover, hydrolyzing the shrimp waste protein for a short period (10 min) without further glycation proved to be useful to produce PEP-inhibiting peptides with potential applications as nootropics. Further work should focus on evaluating the use of the glycated hydrolysates as antioxidant ingredients in food products and their impact on sensory characteristics, as their brown color could limit their application in certain foods.

## Figures and Tables

**Table 1:** Evolution of the degree of hydrolysis along time and content of free amino acids in the hydrolysates before and after 180 min of Maillard glycation. * Indicates significant differences between hydrolysates before and after glycation. The difference in the content of free amino acids before and after glycation is expressed as percentage in parentheses.

Sample	Time of Hydrolysis (min)	DH (%)	Free Amino Acids Content (mEq Ser NH_2_/g Protein)	
			Before Glycation	After Glycation
Control	0	0	1.52 ± 0.07	0.97 ± 0.05 * (−36%)
H10	10	6.09	2.56 ± 0.05	1.33 ± 0.06 * (−48%)
H30	30	10.47	2.43 ± 0.30	3.11 ± 0.09 * (+27%)
H60	60	12.07	3.42 ± 0.05	2.87 ± 0.38 * (−16%)
H120	120	12.08	4.39 ± 0.18	2.71 ± 0.37 * (−38%)

**Table 2:** Molecular weight profile of the protein hydrolysates at different glycation times, measured at different wavelengths (214 nm, 280 nm, 420 nm). The results are expressed in Dalton and correspond to the Mw of the most abundant molecules found in the samples. The relative abundance of each peptide population is indicated in parentheses.

	Glycation Time				
**214 nm**	**0 min**	**40 min**	**90 min**	**150 min**	**180 min**
Control	2836	6455 (8%)	7146 (8%)	6749 (7%)	7065 (7%)
		585 (9%)	656 (8%)	585 (10%)	585 (11%)
		337 (36%)	335 (37%)	335 (40%)	332 (33%)
		194 (47%)	211 (47%)	213 (43%)	222 (50%)
(>1, 1–0.5, <0.5 kDa)	(75, 14, 11%)	(23, 8, 69%)	(26, 8, 67%)	(23, 10, 67%)	(22, 10, 67%)
10 min	949 (24%)	767 (12%)	769 (26%)	772 (13%)	769 (14%)
	574 (29%)	344 (59%)	432 (42%)	343 (59%)	343 (58%)
	279 (28%)	255 (29%)	262 (32%)	254 (29%)	253 (28%)
	<110 (20%)				
(>1, 1–0.5, <0.5 kDa)	(32, 25, 43%)	(23, 15, 62%)	(29, 23, 48%)	(21, 16, 63%)	(22, 16, 61%)
30 min	902 (23%)	748 (12%)	722 (10%)	836 (14%)	713 (12%)
	582 (28%)	342 (57%)	343 (59%)	343 (51%)	332 (58%)
	263 (34%)	239 (33%)	256 (31%)	230 (35%)	250 (30%)
	<110 (15%)				
(>1, 1–0.5, <0.5 kDa)	(25, 28, 46%)	(17, 14, 70%)	(29, 36, 35%)	(33, 34, 33%)	(18, 16, 66%)
60 min	876 (21%)	714 (9%)	693 (10%)	715 (12%)	699 (12%)
	570 (29%)	343 (57%)	343 (56%)	343 (56%)	341 (56%)
	282 (29%)	245 (33%)	246 (34%)	240 (32%)	251 (32%)
	<110 (21%)				
(>1, 1–0.5, <0.5 kDa)	(23, 26, 52%)	(13, 12, 75%)	(14, 13, 73%)	(15, 15, 70%)	(15, 15, 70%)
120 min	857 (21%)	716 (8%)	688 (9%)	693 (22%)	665 (12%)
	565 (32%)	341 (56%)	342 (56%)	437 (42%)	341 (55%)
	274 (28%)	234 (36%)	238 (34%)	254 (36%)	240 (33%)
	<110 (19%)				
(>1, 1–0.5, <0.5 kDa)	(22, 27, 51%)	(10, 11, 79%)	(12, 12, 76%)	(15, 22, 63%)	(14, 15, 71%)
**280 nm**		**40 min**	**90 min**	**150 min**	**180 min**
Control		460 (22%)	480 (15%)	479 (17%)	481 (18%)
		235 (78%)	321 (35%)	328 (37%)	326 (36%)
			241 (50%)	242 (46%)	244 (46%)
(>1, 1–0.5, <0.5 kDa)		(2, 7, 91%)	(5, 9, 87%)	(8, 12, 80%)	(8, 13, 79%)
10 min		342 (60%)	421 (41%)	341 (59%)	341 (60%)
		245 (40%)	247 (59%)	245 (41%)	245 (40%)
(>1, 1–0.5, <0.5 kDa)		(12, 11, 77%)	(18, 17, 65%)	(13, 13, 74%)	(15, 13, 72%)
30 min		341 (61%)	341 (59%)	340 (61%)	339 (60%)
		234 (39%)	245 (41%)	239 (39%)	245 (40%)
(>1, 1–0.5, <0.5 kDa)		(8, 9, 83%)	(9, 10, 81%)	(13, 12, 75%)	(12, 11, 77%)
60 min		341 (58%)	341 (58%)	341 (60%)	339 (60%)
		241 (42%)	244 (42%)	243 (40%)	249 (40%)
(>1, 1–0.5, <0.5 kDa)		(5, 8, 87%)	(7, 10, 83%)	(10, 12, 78%)	(11, 13, 76%)
120 min		339 (58%)	340 (58%)	432 (43%)	339 (61%)
		241 (42%)	242 (42%)	246 (57%)	245 (39%)
(>1, 1–0.5, <0.5 kDa)		(5, 7, 88%)	(6, 10, 84%)	(10, 19, 71%)	(10, 13, 77%)
**420 nm**		**40 min**	**90 min**	**150 min**	**180 min**
Control		496 (20%)	510 (21%)	560 (28%)	551 (26%)
		327 (37%)	358 (32%)		
		281 (43%)	282 (47%)	262 (72%)	277 (74%)
(>1, 1–0.5, <0.5 kDa)		(4, 21, 75%)	(9, 21, 70%)	(14, 22, 64%)	(11, 23, 66%)
10 min					526 (18%)
		291	282	283	286 (82%)
(>1, 1–0.5, <0.5 kDa)		(17, 16, 67%)	(16, 16, 68%)	(18, 16, 66%)	(20, 16, 64%)
30 min				693 (16%)	555 (15%)
		298	284	299 (84%)	280 (85%)
(>1, 1–0.5, <0.5 kDa)		(16, 15, 69%)	(13, 15, 71%)	(21, 15, 64%)	(19, 16, 65%)
60 min				487 (14%)	530 (12%)
		286	283	286 (86%)	280 (88%)
(>1, 1–0.5, <0.5 kDa)		(8, 15, 74%)	(12, 16, 72%)	(15, 15, 70%)	(16, 15, 69%)
120 min				483 (12%)	667 (16%)
		284	281	283 (88%)	281 (84%)
(>1, 1–0.5, <0.5 kDa)		(10, 15, 75%)	(12, 15, 73%)	(13, 15, 71%)	(17, 16, 67%)

**Table 3:** Total phenol (TP, mEq GAE/100 g dry matter), total antioxidant activity as ABTS•+, ORAC, DPPH (µEq Trolox/100 g dry matter), and FRAP (mEq Fe^2+^/100 g dry matter) of the samples before and after different glycation times (0, 40, 90, 150, 180 min).

Hydrolysis/ Heating Time	Assay	0 min	40 min	90 min	150 min	180 min
Control	TP	117.54 ± 0.5 ^Aa^	1375.31 ± 9.64 ^Ac^	1448.11 ± 11.54 ^A^^c^	710.86 ± 0.24 ^Ab^	2405.63 ± 96.95 ^Ad^
H10		432.34 ± 0.2 ^Ba^	1593.26 ± 3.35 ^Bb^	4220.61 ± 127.81 ^B^^c^	3553.15 ± 65.62 ^Bc^	11,161.73 ± 6.85 ^Bd^
H30		640.81 ± 8.33 ^Da^	3851.77 ± 124.01 ^Eb^	4144.05 ± 137.38 ^B^^b^	5808.61 ± 85.36 ^Ec^	9718.77 ± 63.36 ^Cd^
H60		588.3 ± 5.7 ^Ca^	2723.46 ± 37.31 ^Cb^	4951.76 ± 16.57 ^C^^c^	4518.19 ± 104.63 ^Dc^	10,875.64 ± 17.91 ^Cd^
H120		585.9 ± 4.1 ^Ca^	3214.33 ± 96.06 ^Db^	5034.13 ± 43.66 ^Cd^	4053.64 ± 62.00 ^Cc^	12,865.95 ± 487.75 ^De^
Control	ABTS•+	34.0 ± 6.2 ^Aa^	425.8 ± 8.5 ^Ac^	421.6 ± 3.7 ^Ac^	211.4 ± 0.6 ^Ab^	3045.18 ± 25.2 ^Ad^
H10		107.3 ± 23.1 ^Ba^	890.7 ± 18.2 ^Bb^	1235.1 ± 80.0 ^BCc^	1083.5 ± 21.2 ^Bc^	9749.84 ± 75.7 ^Bd^
H30		188.4 ± 12.3 ^Ca^	1163.3 ± 55.7 ^Cb^	1122.8 ± 104.3 ^Bb^	1601.1 ± 65.1 ^Cc^	9256.53 ± 34.8 ^Bd^
H60		111.4 ± 1.6 ^Ba^	742.9 ± 7.5 ^Db^	804.5 ± 109.6 ^Db^	1136.3 ± 133.1 ^Bb^	10,090.7 ± 62.1 ^Cc^
H120		178.3 ± 2.8 ^Ca^	936.6 ± 17.8 ^Bb^	1358.9 ± 81.6 ^Cc^	902.8 ± 150.5 ^Bd^	11,126.3 ± 64.9 ^Dd^
Control	ORAC	6.56 ± 0.28 ^Aa^	79.10 ± 6.6 ^Abc^	102.68 ± 6.17 ^Ac^	74.50 ± 1.2 ^Ab^	411.5 ± 18.8 ^Ad^
H10		62.23 ± 4.35 ^Ba^	478.19 ± 8.9 ^Db^	537.47 ± 10.98 ^Bc^	521.09 ± 32.6 ^Bc^	1966.0 ± 46.8 ^Cd^
H30		94.73 ± 6.44 ^Ca^	517.28 ± 18.78 ^Eb^	517.66 ± 16.46 ^Bb^	1066.95 ± 35.0 ^Dc^	1827.4 ± 17.2 ^Bd^
H60		84.83 ± 7.11 ^BCa^	359.50 ± 24.41 ^Bb^	654.81 ± 133.33 ^Bc^	632.92 ± 30.3 ^Cc^	1983.5 ± 14.9 ^Cd^
H120		70.55 ± 7.15 ^Ba^	450.86 ± 7.21 ^Cb^	871.75 ± 65.67 ^Cd^	619.58 ± 29.3 ^Cc^	2105.1 ± 40.2 ^De^
Control	DPPH	0.02 ± 0.00 ^Aa^	0.12 ± 0.00 ^Ad^	0.06 ± 0.00 ^Ac^	0.00 ± 0.00 ^A^^b^	0.12 ± 0.00 ^Ad^
H10		0.06 ± 0.00 ^Aa^	0.11 ± 0.00 ^Bb^	0.25 ± 0.00 ^Cc^	0.24 ± 0.01 ^D^^c^	0.41 ± 0.01 ^BCd^
H30		0.03 ± 0.00 ^Aab^	0.35 ± 0.01 ^Cab^	0.37 ± 0.02 ^Eb^	0.28 ± 0.01 ^E^^a^	0.40 ± 0.00 ^Bc^
H60		0.07 ± 0.01 ^Aa^	0.24 ± 0.00 ^Dc^	0.29 ± 0.01 ^Dd^	0.22 ± 0.02 ^C^^b^	0.43 ± 0.00 ^Ce^
H120		0.06 ± 0.00 ^Aa^	0.27 ± 0.00 ^Ed^	0.22 ± 0.00 ^Bc^	0.19 ± 0.00 ^B^^b^	0.46 ± 0.01 ^De^
Control	FRAP	0.03 ± 0.00 ^Aa^	0.39 ± 0.02 ^Ac^	0.52 ± 0.01 ^Ad^	0.18 ± 0.00 ^A^^b^	1.22 ± 0.02 ^Ae^
H10		0.07 ± 0.00 ^Ba^	0.67 ± 0.012 ^Bb^	1.89 ± 0.10 ^Dc^	0.80 ± 0.01 ^Bb^	2.05 ± 0.06 ^Bd^
H30		0.13 ± 0.00 ^Ca^	0.84 ± 0.02 ^Cc^	0.77 ± 0.01 ^Bb^	1.27 ± 0.00 ^Ed^	2.51 ± 0.02 ^De^
H60		0.20 ± 0.00 ^Ca^	0.58 ± 0.00 ^Db^	1.08 ± 0.00 ^Cd^	1.01 ± 0.005 ^Dc^	2.25 ± 0.01 ^Ce^
H120		0.12 ± 0.00 ^Da^	0.53 ± 0.00 ^Eb^	1.18 ± 0.00 ^Cd^	0.92 ± 0.05 ^Cc^	2.80 ± 0.09 ^Ee^

For each marker. Values (mean ± standard deviation, *n* = 3) followed by the same uppercase letter in same column are not significantly different (*p* < 0.05). Values (mean ± standard deviation, *n* = 3) followed by the same lowercase letter in the same row, for each parameter, are not significantly different (*p* < 0.05).

## Data Availability

Data sharing is not applicable to this article.
